# Coevolution of social and communicative complexity in lemurs

**DOI:** 10.1098/rstb.2021.0297

**Published:** 2022-09-26

**Authors:** Claudia Fichtel, Peter M. Kappeler

**Affiliations:** ^1^ Behavioral Ecology and Sociobiology Unit, German Primate Center, Leibniz Institute for Primate Research, Kellnerweg 4, Göttingen 37077, Germany; ^2^ Leibniz-ScienceCampus Primate Cognition, Kellnerweg 4, 37077 Göttingen, Germany; ^3^ Department Anthropology/Sociobiology, University of Göttingen, Kellnerweg 6, 37077 Göttingen, Germany

**Keywords:** social complexity, communicative complexity, primates, vocalizations, olfaction, visual signals

## Abstract

The endemic lemurs of Madagascar (Lemuriformes: Primates) exhibit great social and communicative diversity. Given their independent evolutionary history, lemurs provide an excellent opportunity to identify fundamental principles in the coevolution of social and communicative traits. We conducted comparative phylogenetic analyses to examine patterns of interspecific variation among measures of social complexity and repertoire sizes in the vocal, olfactory and visual modality, while controlling for environmental factors such as habitat and number of sympatric species. We also examined potential trade-offs in signal evolution as well as coevolution between body mass or brain size and communicative complexity. Repertoire sizes in the vocal, olfactory and visual modality correlated positively with group size, but not with environmental factors. Evolutionary changes in social complexity presumably antedated corresponding changes in communicative complexity. There was no trade-off in the evolution of signals in different modalities and neither body mass nor brain size correlated with any repertoire size. Hence, communicative complexity coevolved with social complexity across different modalities, possibly to service social relationships flexibly and effectively in pair- and group-living species. Our analyses shed light on the requirements and adaptive possibilities in the coevolution of core elements of social organization and social structure in a basal primate lineage.

This article is part of the theme issue ‘Cognition, communication and social bonds in primates’.

## 1. Introduction

A major goal in evolutionary biology is to explain the astonishing diversity of animal signals, characterized by great variation in both the nature and number of signals used for communication. Despite the acknowledged role of multiple factors, including phylogenetic history, genetic or cultural drift, environmental factors and sexual selection [1,2], the social complexity hypothesis for communicative complexity (SCHCC) emerged as a prominent additional explanation for signal diversification. The SCHCC proposes that animals living in groups with comparatively greater social complexity also exhibit greater complexity in their communicative systems [1–3]. Living in groups entails repeated interactions among different individuals, requiring the ability to assess the behaviour of others and to respond flexibly and adaptively to it [4–6]. The required social competence has been suggested to fuel the need for increasingly complex signals because they are the key mechanism mediating these interactions [3,7–9].

Several studies of diverse taxa, ranging from insects to primates, have provided support for the SCHCC. In primates, species living in larger groups have larger vocal repertoires [7] and exhibit more facial expressions [10]. Variation in primate social structure, e.g. in the prevalence of a particular dominance style or inter-sexual bonds, also covaries with a larger vocal repertoire in either dominance- or affiliation-related vocalizations, respectively [11–13]. Even within species, as for example, in chimpanzees (*Pan troglodytes schweinfurthii*), variation in social complexity is associated with variation in gesture use [14]. In lemurs, variation in social organization and inter-sexual dominance style is associated with complexity of scent compositions in glandular secretions [15]. Similar relationships have also been described in other taxa. For example, in bats, the information content of vocalizations is positively related to the relevant social group size [16]. Similarly, in sciurid rodents, demographic role complexity predicts alarm call repertoire size [17], and group size predicts alarm call individuality [18]. In birds, cooperative breeders exhibit larger vocal repertoires [19,20], experimental manipulations of group size caused changes in song complexity [21] and group-living facilitated duetting [22]. In lizards, social grouping covaries positively with the number of chemical signalling glands [23], whereas the visual display repertoire size is inversely associated with home range size [24]. Finally, in insects, sensory systems and chemical signals coevolved with group-living in halictid bees [25]. Although it is notoriously challenging to operationalize both social and communicative complexity [2,26–31], the SCHCC has enjoyed support from studies using a range of proxies for social and communicative complexity.

However, the majority of studies investigating the SCHCC considered only one communicative modality at a time [1,2,32]. Investigating one communicative modality only can result in an over- or underestimation of communicative complexity because behavioural traits can be expressed in different modalities. In primates, for example, submission can be expressed either by visual or vocal signals [33,34]. In addition, alternative explanations for signal diversification, such as morphological constraints governing signal production, cultural or genetic drift, or environmental factors ought to be considered [32]. For example, the production of acoustic signals across 500 animal species is primarily controlled by individual metabolism, such that basic acoustic features vary with body mass and temperature [35], even within species [36]. Body size can also correlate with the evolution of colourful visual signals in insects or visual display repertoires in lizards [37–39]. In addition, signal diversification can also be a result of cultural or genetic drift [40–42] or environmental factors [8,32]. For example, background noise can either limit the range of signals expressed, reducing signal complexity [43,44], or facilitate the evolution of new components to signals attracting the attention of receivers, thereby increasing signal complexity [45].

Finally, social factors emerging not only from conspecifics, but also from sympatric heterospecifics may trigger the evolution of complex signals [32]. In New and Old World monkeys, for example, interspecific variation in facial colour patterns covaries with the number of species living in sympatry. Species living with more sympatric congeners exhibit more complex facial colour patterns than species without or with fewer sympatric congeners, possibly to reduce the risk of hybridization [46,47]. Variation in facial hair length and colour, however, was related to environmental factors, with species living in cooler areas having longer facial hairs and species living in denser forests exhibiting darker facial colours than species in more open habitats. Interestingly, in lemurs, facial colour complexity was not related to the number of sympatric heterospecifics, most likely because members of only two genera occur in sympatry with congeners. However, similar to New and Old World monkeys, facial hair length and colour covaried with environmental factors [48], highlighting the notion of also considering alternative explanations for signal diversification. Thus, additional comprehensive tests of the SCHCC are indicated, in particular in studies that consider multiple modalities.

Lemurs (Lemuriformes), an adaptive radiation of primates endemic to Madagascar, provide an excellent opportunity for an independent test of the SCHCC because they evolved in isolation from other primates for more than 50 Myr [49] and group-living has evolved twice independently [50]. With currently more than 120 recognized species, lemurs are taxonomically diverse and exhibit all major forms of social organization, i.e. solitary, pair- and group-living, found among anthropoid primates [51,52]. They live in habitats that they share with a range of different species exhibiting similar activity patterns, and with at least one species belonging to the same family, whereas only two genera occur in sympatry with a maximum of one congener per location [48]. They also vary in activity patterns, including diurnal, cathemeral and nocturnal species [53], which may affect the evolution of visual signals in nocturnal or cathemeral species due to visual constraints at night. Activity patterns also covary with social organization and morphology, with nocturnal species being solitary or pair-living but also smaller and more cryptic, most likely as an adaptation to predation pressure [54,55]. Lemurs occupy different forest types, ranging from dry to humid forests, with either an open or closed canopy structure, impacting signal transmission more or less [56]. Most importantly, lemurs exhibit diversity in vocalizations that is unique among primates, with some species producing calls that range into the ultra-sound range. Moreover, lemurs exhibit a spectacular diversity of olfactory signals and use different sources to produce scent-marks, ranging from saliva, faeces and urine to a variety of specialized glands [57,58]. In the domain of visual signals, they exhibit a range of facial colour patterns [48], but they also use manual or bodily gestures as well as facial expressions, which are, for obvious reasons, less well studied in nocturnal lemurs.

The aim of this study was to investigate the relative importance of social and ecological factors as well as morphology in shaping the repertoire size in vocal, olfactory and visual signals in lemurs. We used group size as our social factor to operationalize social complexity because all other measures of social complexity are positively related to it [28]. As socio-ecological factors, we used the number of sympatric species during the respective activity period of a given species (nocturnal, cathemeral, diurnal) and the number of congeners because the presence of community members producing similar signals and the resulting need for reliable species recognition may promote signal diversification [59,60]. As an ecological factor, we studied variation in habitat type, distinguishing regions with semi-open or closed canopy structure, because habitat density may impact signal transmission across modalities [61].

Since signals can be costly to produce, we additionally investigated whether there is a trade-off in the evolution of signals across modalities, i.e. whether investment in the evolution of signals in one modality comes at the expense of the evolution of signals in another modality [62]. Finally, we investigated whether the evolution of larger vocal or visual repertoires covaries with evolutionary changes in brain size. Many signals are used to establish and maintain social relationships between group members, and being able to reliably assess the behaviour of others and to respond adaptively to it is a key cognitive ability [4,8,31]. Since primates living in larger groups have more differentiated social relationships and larger brains for their body size [31], the number of signals employed to mediate social relationships might be related to cognitively complex communication strategies [8,14,63].

## 2. Methods

### (a) Data compilation

We extracted data on vocal repertoire size, number of sources of scent-marks and number of visual signals from the literature (electronic supplementary material, table S1). For the selection of vocal repertoire sizes, we followed the criteria of McComb & Semple [7] as closely as possible. Since in some species different studies revealed different vocal repertoire sizes (electronic supplementary material, table S1), we conducted statistical analyses with minimum, mean or maximum vocal repertoire size, respectively. As sources of olfactory signals, we collected data on whether a species uses saliva, urine or faeces for this specific purpose and/or we determined the number of different glands with which they produce scent-marks (electronic supplementary material, table S1). We defined visual signals as body postures, gestures and facial expressions (electronic supplementary material, table S1). As socio-ecological factors (electronic supplementary material, table S2), we included information on group size [50], and the number of sympatric species with the same activity period (nocturnal, cathemeral, diurnal) or that belong to the same genus [56]. We collected information on habitat type and the number of sympatric species based on their distribution, using the classification by Muldon & Goodman [56]. Spiny thicket, succulent woodland and dry deciduous forests were classified as semi-open habitats and subhumid and humid forests as closed habitats. Information on endocranial volume (ECV) and body mass were taken from [50,64].

### (b) Statistical analyses

We fitted several phylogenetic generalized least-squares regressions (PGLSs), using a Brownian motion model, to estimate whether communicative complexity is predicted by group size, the number of sympatric species with the same activity or from the same genus, or habitat types, while correcting for phylogenetic uncertainty using the packages ‘ape’ [65], ‘caper’ [66] and ‘geiger’ [67] in R. v. 4.0.3 [68]. We calculated a consensus tree based on 100 phylogenetic trees obtained from vertlife.org [69] to represent the evolutionary history of these species and its uncertainty. Each model was fitted first on the consensus tree (electronic supplemental material, figure S1) and then on each of 100 phylogenetic trees. We checked the assumptions of normality distributions and homogeneity by visual inspection of a QQ-plot of residuals and residuals plotted against fitted values for models fitted with the consensus tree [70]. Phylogenetic signal was estimated by Pagel's lambda using maximum likelihood. In addition, likelihood ratio tests were used to calculate *p*-values assessing whether the estimated maximum likelihood value of lambda differs significantly from 0 or 1.

Model statistics for the results of the PGLS based on the consensus tree are reported in §3 and estimates and standard errors for the results of the PGLSs from each of the 100 trees are reported in the electronic supplementary material. Since group size covaries with social organization and activity patterns (electronic supplementary material, tables S2 and S3), we included only group size as a proxy for sociality.

(1) For vocal repertoire sizes, we calculated three sets of models by using either mean, minimum or maximum vocal repertoire size as response variable. Because vocal repertoire size can, depending on the methods used to classify call types, vary across studies for a given species, we used mean, minimum or maximum vocal repertoire size to acknowledge this variation. Variation in repertoire size was not influenced by sampling effort (negative binomial model: mean repertoire: estimate: 0.12, s.e.: 0.09, *p* = 0.200; minimum repertoire: estimate: −0.05, s.e.: 0.10, *p* = 0.630; maximum repertoire: estimate: 0.16, s.e.: 0.10, *p* = 0.100). As fixed factors, we included either group size, habitat type (semi-open, closed), number of sympatric species with the same activity pattern or the number of congeners; both values were log- and z-transformed. (2) We estimated whether the number of olfactory sources is predicted by either group size, habitat type, number of sympatric species with the same activity pattern or from the same genus. Since data were compiled from one review and/or several studies focusing on single sources of scent production, we did not control for sampling effort. (3) For visual signals, we found information for only ten species. Therefore, we fitted several models with the number of visual signals as response and only one predictor as fixed factor at a time, i.e. either (i) group size, (ii) habitat type, (iii) the number of sympatric species with the same activity pattern or (iv) from the same genus. Since the visual repertoire size was compiled only from single studies, we did not control for sampling effort.

(4) We fitted six evolutionary models of character evolution for continuous data to estimate whether changes in social organization (solitary, pair- and group-living) were associated with selective constraints on the evolution of the mean vocal repertoire size and number of olfactory sources but not on the number of visual signals due to the small sample size. Models differed in how they allow the trait of interest (social organization) to influence the rate, optima and/or strength of selection towards the optima or to have no impact on the continuous trait (number of signals). The BMI model assumes no difference between the types of social organization and that the number of signals, therefore, evolve according to a Brownian motion process. The BMS model assumes that the number of signals evolves at different rates for each type of social organization. The OUM model allows the number of signals to evolve with different optima but identical strength of selection and with a rate of stochastic motion acting on all types of social organization. The OUMA model allows only the strength of selection to vary across social organization types, whereas the OUMVA model allows all three parameters to vary among the different types of social organizations. Since these models are sensitive to small sample sizes [71], we report only the model fits but not the values for the rate, strength of selection or the phenotypic optima, because these parameters might be estimated incorrectly.

(5) To investigate whether the production of more signals in one modality comes at a cost for signal production in another modality, we calculated several PGLS models fitting mean, minimum, maximum vocal repertoire size as response and (i) either the number of olfactory sources or (ii) visual signals as fixed factor and (iii) the number of olfactory sources as response and the number of visual signals as fixed factor. (6) Finally, to examine whether communicative complexity covaries with cognitive abilities, we calculated two more models using either mean vocal repertoire size or the number of visual signals as response and as fixed factors the log transformed ECV and body mass, respectively. Since results did not differ in previous models in relation to mean, minimum and maximum vocal repertoire size, we used only mean vocal repertoire size for these analyses. For the number of olfactory sources, we only investigated whether it covaries with body mass (log transformed) because the evolution of olfactory sources might rather be phylogenetically constrained and not related to variation in brain size.

Because some models suggested no or weak phylogenetic signal, indicated by Pagel's lambda, we additionally fitted non-phylogenetic linear models with a Poisson distribution and report them in the electronic supplemental material.

## 3. Results

### (a) Vocal communication

**Figure 1.**
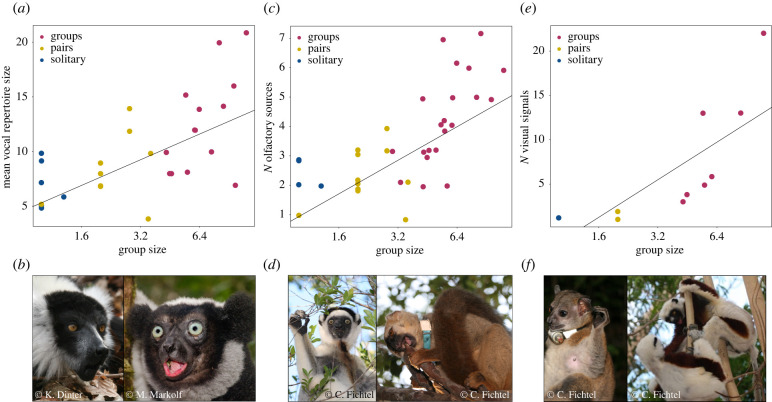
Relationships between communicative and social complexity across modalities. (*a*) Mean repertoire size as function of group size. (*b*) Photos depict a black-and-white ruffed lemur and an indri vocalizing. (*c*) Number of sources for olfactory signals as a function of group size. (*d*) A male Verreaux's sifakas with a chest gland (brown spot on the chest) and a redfronted lemur depositing a scent-mark with saliva while chewing on a branch, wearing a neck collar to facilitate individual recognition. (*e*) Number of visual signals as a function of group size. (*f*) A redtailed sportive lemur displaying the faint-to-cuff gesture, wearing a radio-collar and two Coquerel's sifakas displaying the open-mouth facial expression while playing. Lines indicate regression lines of (*a*) model table 1b, (*c*) model table 2b and (*e*) model table 3b. Social organization is indicated by colours, with solitary species = blue, pair-living species = yellow and group-living species = red. *X*-axis labels are back-transformed to original values.

Vocal repertoire sizes were available for 29 species (electronic supplementary material, table S1). The average mean vocal repertoire size was 10 ± 4 call types (mean ± s.d.: minimum repertoire size = 9 ± 4; maximum repertoire size = 11 ± 5). The mean vocal repertoire size correlated positively with group size, but not with habitat type or the number of sympatric species with the same activity pattern or from the same genus (table 1a,b; electronic supplementary material, S4). Lemurs living in larger groups evolved larger vocal repertoires (figure 1*a*). The estimated fits of the models looped over the 100 phylogenetic trees were very stable and similar to the fit of the model based on the consensus tree (electronic supplementary material, figures S2 and S3). The minimum and maximum vocal repertoire sizes were also predicted by group size, but not by habitat type and the number of sympatric species with the same activity pattern or from the same genus (electronic supplementary material, tables S5a–d and S6a–d). Model results were also stable across the 100 phylogenetic trees and comparable to the fit of the models based on the consensus tree (electronic supplementary material, figures S4–S7).

**Table 1.** Mean vocal repertoire size (*N* = 29 species). Results of the PGLSs on the influence of group size, habitat type (semi-open, closed) and (*a*) the number of sympatric species with the same activity pattern or (*b*) the number of sympatric congeners on the mean vocal repertoire size.

model	term	estimate	s.e.	*p*-value
(*a*) mean vocal repertoire size	intercept	5.56	1.53	^b^
	group size	0.84	0.24	0.002
	habitat type (semi-open)^a^	1.03	1.24	0.415
	*N* sympatric species with same activity	0.01	0.06	0.848
*lambda =* 0.196; 0: *p =* 0.311; 1: *p <* 0.001				
(*b*) mean vocal repertoire size	intercept	5.14	1.33	^b^
	group size	0.93	0.23	<0.001
	habitat type (semi-open)^a^	1.06	1.23	0.396
	*N* sympatric congeners	0.50	0.39	0.213
*lambda =* 0.066; 0: *p =* 0.768; 1: *p <* 0.001				

^a^Open habitat as reference category.

^b^Not shown as has no meaningful interpretation.

### (b) Olfactory communication

We found information on olfactory sources to produce scent-marks for 37 species with a mean ± s.d. number of 3 ± 2 olfactory sources (electronic supplementary material, table S1). The PGLS based on the consensus tree revealed that the number of olfactory sources correlated positively with group size: lemurs living in larger groups used more olfactory sources to produce scent-marks (figure 1*c*; table 2*a*,*b*; electronic supplementary material, S7). Habitat type influenced the number of olfactory sources with species occurring in semi-open habitats exhibiting a higher number of olfactory sources. The number of sympatric species with the same activity pattern or from the same genus did not influence variation in the number of olfactory sources. The estimated fits of the models looped over the 100 phylogenetic trees varied little and were similar to the fit of the models based on the consensus tree (electronic supplementary material, figures S8 and S9).

**Table 2.** Olfactory sources (*N* = 37 species). Results of the PGLSs on the influence of group size, habitat type (semi-open, closed) and (*a*) the number of sympatric species with the same activity pattern or (*b*) the number of congeners on the number of olfactory sources to produce scent-marks.

model	term	estimate	s.e.	*p*-value
(*a*) number of olfactory sources	intercept	2.87	0.42	^b^
	group size	1.22	0.27	<0.001
	habitat type (semi-open)^a^	0.74	0.35	0.042
	*N* sympatric species with same activity	0.09	0.19	0.609
*lambda* *=* 0.524*;* 0: *p* *=* 0.001*;* 1*: p* *<* 0.001				
(*b*) number of olfactory sources	intercept	2.94	0.34	^b^
	group size	1.26	0.25	<0.001
	habitat type (semi-open)^a^	0.78	0.34	0.027
	*N* sympatric congeners	0.34	0.18	0.063
*lambda* *=* 0.357*;* 0: *p* *=* 0.119*;* 1: *p* *<* 0.001				

^a^Open habitat as reference category.

^b^Not shown as has no meaningful interpretation.

### (c) Visual communication

We found information on the number of visual signals for only ten species (electronic supplementary material, table S1). In nocturnal lemurs, one solitary and two pair-living species had one to two visual signals, whereas the cathemeral or diurnal group-living lemurs exhibited 3–22 visual signals. Based on the consensus tree, the number of visual signals was predicted by group size, but not by habitat type or the number of sympatric species with the same activity pattern or from the same genus (figure 1*e*; table 3*a*–*d*; electronic supplementary material, S8). The estimated fits of the models looped over 100 phylogenetic trees were comparable to the estimates of the consensus tree (electronic supplementary material, figures S10–S13).

**Table 3.** Visual signals (*N* = 10 species). Results of the PGLSs on the influence of (*a*) group size, (*b*) habitat type (semi-open, closed) and (*c*) the number of sympatric species with the same activity pattern or (*d*) the number of congeners on the mean number of visual signals.

model	term	estimate	s.e.	*p*-value
(*a*) number of visual signals	intercept	5.56	2.42	^b^
	group size	4.52	1.71	0.029
*lambda* *=* 0.933; 0: *p* *=* 0.076; 1: *p* *=* 0.521				
(*b*) number of visual signals	intercept	5.44	3.48	^b^
	habitat (semi-open)^a^	0.83	1.49	0.596
*lambda* *=* 1; 0: *p* *=* 0.003; 1: *p* *=* *1*				
(*c*) number of visual signals	intercept	5.96	3.35	^b^
	*N* sympatric species with same activity	−0.52	0.96	0.602
*lambda* *=* 1; 0: *p* *=* 0.003; 1: *p* *=* 1				
(*d*) number of visual signals	intercept	5.99	3.42	^b^
	*N* sympatric congeners	−0.07	0.67	0.923
*lambda* *=* 1; 0: *p* *=* 0.004; 1: *p* *=* 1				

^a^Open habitat as reference category.

^b^Not shown as has no meaningful interpretation.

### (d) Evolutionary model fitting

The OUMVA model received the strongest support for the evolution of the mean number of vocal signals and the number of olfactory sources (table 4), suggesting that social organization influenced all parameters (optimum, rate of stochastic motion and strength of selection) for the evolution of these signals. However, due to the small sample size these results should be interpreted with caution.

**Table 4.** AICc and ΔAICc representing the relative likelihood of each of the six evolutionary models investigating if social organization influences the evolution of mean vocal repertoire size and the number of olfactory sources.

models	mean vocal repertoire size		no. olfactory sources	
	AICc	ΔAICc	AICc	ΔAICc
BMI	176.00	22.45	141.01	32.55
BMS	176.03	44.48	141.20	32.74
OU1	173.88	20.33	137.63	29.17
OUM	178.44	24.89	137.23	28.77
OUMA	157.08	3.53	112.79	4.33
OUMVA	153.55	0	108.46	0

### (e) Trade-offs in signal evolution across modalities

**Figure 2.**
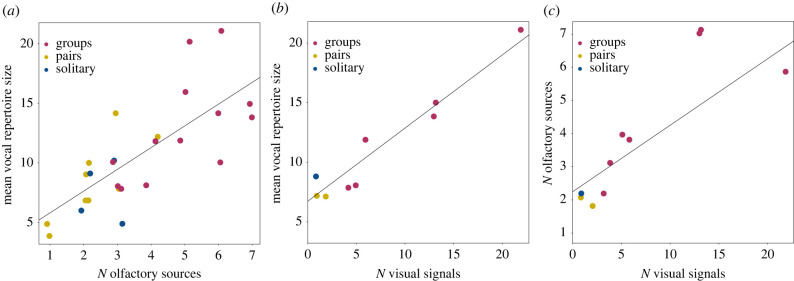
Relationship between (*a*) mean vocal repertoire size and the number olfactory sources and (*b*) the number of visual signals as well as (*c*) the number olfactory sources and visual signals. Lines indicate the regression lines. Activity patterns are indicated by colours, with blue indicating solitary species, yellow indicating pair-living species and red indicating group-living species.

Vocal repertoire sizes (mean, minimum, maximum) correlated positively with the number of olfactory sources and visual signals (figure 2*a–c* and table 5*a*–*f*; electronic supplementary material, S9). Also, the number of olfactory sources covaried positively with the number of visual signals (table 5*g*). For all models, the estimated fits of the models looped over the 100 phylogenetic trees were very stable and similar to the fit of the model based on the consensus tree (electronic supplementary material, figures S14–S20). Hence, the evolution of more signals in one modality did not come at the expense of the evolution of signals in another modality, suggesting that if more signals were required to mediate social interactions they evolved in all communicative modalities.

**Table 5.** Results of the PGLS on the relationships between (*a*–*f*) vocal repertoire sizes (mean, minimum, maximum) and the number of olfactory sources or the number of visual signals and (*g*) the number of olfactory sources and the number of visual signals.

model	term	estimate	s.e.	*p*-value
(*a*) mean vocal repertoire size	intercept	3.97	1.35	^a^
*N* = 26	*N* olfactory sources	1.83	0.33	<0.001
*lambda* *=* 0; 0: *p* *=* **1; 1:* p* *<* *0.001*				
(*b*) mean vocal repertoire size	intercept	6.74	0.80	^a^
*N* = 9	*N* visual signals	0.61	0.07	<0.001
*lambda* *=* 0.820; 0: *p* *=* 0.220; 1: *p* *<* 0.091				
(*c*) minimum vocal repertoire size	intercept	4.50	1.48	^a^
*N* = 26	*N* olfactory sources	1.41	0.37	<0.001
*lambda* *=* 0; 0: *p* *=* 1; 1: *p* *<* 0.001				
(*d*) minimum vocal repertoire size	intercept	6.33	0.81	^a^
*N* = 9	*N* visual signals	0.58	0.08	<0.001
*lambda* = 0; 0: *p* = 1; 1: *p* *<* 0.001				
(*e*) maximum vocal repertoire size	intercept	3.65	1.39	^a^
*N* = 26	*N* olfactory sources	2.06	0.34	<0.001
*lambda* = 0; 0: *p* = 1; 1: *p* *<* 0.001				
(*f*) maximum vocal repertoire size	intercept	6.84	0.93	^a^
*N* = 9	*N* visual signals	0.67	0.09	<0.001
*lambda* = 0.613; 0: *p* = 0.355; 1: *p* = 0.008				
(g) *N* olfactory sources	intercept	2.25	0.91	^a^
*N* = 10	*N* visual signals	0.20	0.08	0.037
*lambda* = 1; 0: *p* = 0.639; 1: *p* = 1				

^a^Not shown as has no meaningful interpretation.

### (f) Morphology and brain size

Neither the mean vocal repertoire size nor the number of visual signals covaried with ECV or body mass (table 6*a*,*c*; electronic supplementary material, S10). The number of olfactory sources and visual signals did not correlate with body mass (table 6*b*; electronic supplementary material, S10). The estimated fits of the models looped over the 100 phylogenetic trees varied little and were comparable to the fit of the models based on the consensus tree (electronic supplementary material, figures S21–S23).

**Table 6.** Results of the PGLSs on the influence of ECV and body mass in their interaction on (*a*) the mean vocal repertoire size and (*b*) body mass on the number of olfactory sources or (*c*) visual signals.

model	term	estimate	s.e.	*p*-value
(*a*) mean vocal repertoire size	intercept	−4.79	11.76	^a^
	ECV	−2.48	4.10	0.553
*N* = 23	body mass	2.92	3.04	0.358
*lambda* = 0.487; 0: *p* = 0.035; 1: *p* = 0.001				
(*b*) number of olfactory sources	intercept	3.00	0.93	^a^
*N* = 36	body mass	0.53	0.34	0.130
*lambda* = 0.576; 0: *p* *<* 0.001; 1: *p* *<* 0.001				
(*c*) number of visual signals	intercept	11.51	3.11	^a^
	ECV	27.57	16.45	0.155
*N* = 8	body mass	−28.87	18.99	0.189
*lambda* = 0; 0: *p* = 1; 1: *p* = 1				

^a^Not shown as has no meaningful interpretation.

## 4. Discussion

Our study revealed that variation in the vocal, olfactory and visual repertoire size of lemurs covaried with group size, but not with the number of sympatric species with the same activity pattern or from the same genus, or environmental factors such as habitat type, except for the number of olfactory sources. We did not find evidence for a trade-off in the evolution of signals, because lemurs that evolved more signals in one modality also did so in another modality. Communicative complexity in vocal and visual signals did not covary with brain size (ECV) or morphology. Hence, by controlling for alternative explanation, we could show that communicative complexity in all three modalities coevolved with social complexity, and the coevolution models suggest that communicative complexity changed in response to evolutionary changes in social complexity.

### (a) Social complexity

The number of vocal or visual signals, as well as the number of olfactory sources, correlated positively with group size. Although group size has been criticized as an appropriate proxy for social complexity, it nevertheless defines one cornerstone of social complexity. In addition, group size should be a meaningful proxy of social complexity because the number of individuals an animal can interact with covaries with group size [28]. However, complexity in social interactions, such as grooming, may better reflect social complexity in species with individual recognition and repeated interactions, such as primates [28,72]. Unfortunately, data on social interaction rates are rare for lemurs, especially in nocturnal species, currently limiting comparative studies to the use of group size as the best available proxy for social complexity in those species for which information on repertoire size exists.

### (b) Communicative complexity

#### (i) Vocal communication

All measures of vocal repertoire sizes correlated with group size, supporting earlier findings on the coevolution of vocal and social complexity across all primates [7,11–13]. Our analyses also suggest that changes in social organization influenced the evolution of diversity in vocal signals, but these results should be interpreted carefully because of the small sample size. Lemurs are considered as living models of ancestral primates and, although they live in smaller groups than anthropoid species of the same body size [73], their vocal communication is in many respects as elaborate as in anthropoid primates. They use vocalizations to mediate social interactions [74–76], to signal dominance status [33,74], to warn off or to deter predators, including functionally referential alarm calls [77–81], to coordinate group movements and to maintain group cohesion [82–85], to attract mates or to advertise individual quality [86–89], to defend territories [90] or for species recognition [91].

In addition, vocal exchanges of contact calls mirror social relationships, with ring-tailed lemurs (*Lemur catta*) responding selectively only to contact calls of group members with whom they have a strong social relationship [92]. Contact calls may, hence, serve to maintain a social bond [92]. There is also evidence that the perception of signals and cues is related to social complexity [93]. In lemurs and other strepsirrhines, adaptations to perceive acoustic signals with enhanced hearing sensitives coevolved with group size [94]. Hence, sustained social interactions might have been facilitated by the ability to signal flexibly and to perceive signals and cues of others effectively [2,3,6,93,95].

However, assessing vocal repertoire size correctly is challenging, because the number of identified call types depends on the underlying clustering procedure applied to categorize call types, in particular of calls exhibiting graded variants between call types [2,27]. As a consequence, vocal repertoire sizes reported by different studies can vary strikingly for a given species. For example, in black lemurs (*Eulemur macaco*), the reported vocal repertoire sizes varied from 7 to 16 call types [96–99]. Repeating analyses with the mean, minimum and maximum vocal repertoire sizes should circumvent this fundamental problem until the experts agree on a particular repertoire size and revealed robust patterns, irrespective of the particular values used.

#### (ii) Olfactory communication

The number of olfactory sources also covaried with social complexity, suggesting that even this rather rough measure of different sources to produce scent-marks correlates with social complexity. Evolutionary models also suggested that changes in social organization have influenced the evolution of the number of olfactory sources. Moreover, independent of whether olfactory signals are based on saliva, faeces or glandular secretions, they appear to offer rich sources of information. Urine marking, for example, has been suggested to be an ancestral behaviour related to a nocturnal, solitary lifestyle, whereas the various forms of glandular marking evolved in parallel, and represent derived states [100]. Nocturnal lemurs also evolved fewer scent glands than cathemeral or diurnal lemurs (electronic supplementary material, table S2). Urine consists of fewer (2–13) olfactorily relevant compounds, in comparison to glandular secretions, which consist of up to 300 compounds [100,101]. Deposition of urine marks is also rather discrete and might be designed for delayed olfactory detection [100]. Although some glandular markings may also be designed for delayed detection, they are often accompanied by conspicuous body postures, constituting a multimodal signal, facilitating rapid visual detection, olfactory inspection or even countermarking by group members [2,101,102]. Hence, with the transition to a diurnal and more gregarious lifestyle, (additional) glandular secretions might have evolved to facilitate intra- but also inter-group communication [100], allowing generally more complex communicative strategies.

In addition, the chemical composition of glandular secretions covaries with measures of social complexity, as for example, social organization and dominance style in eulemurs [15]. Hence, the basic morphological hardware to produce olfactory signals but also the composition of the various secretions coevolved with social complexity in lemurs. Similarly, in lizards the basic morphological hardware to produce olfactory signals, i.e. the number of chemical signalling glands, correlated with social grouping [23], suggesting that this rather rough proxy of communicative complexity is sufficient to invoke convergent coevolution with social complexity.

#### (iii) Visual communication

The number of visual signals also correlated positively with increasing group size. Visual signals in lemurs are generally less well studied, particularly in nocturnal species. As a consequence, we found information on visual signals for only ten lemur species. Among nocturnal species, the solitary grey mouse lemurs (*Microcebus murinus*) and pair-living redtailed and Sahamalaza sportive lemurs (*Lepilemur ruficaudatus* and *L. sahamalenzis*, respectively) exhibit a feint-to-cuff gesture (figure 1*b*), where individuals rapidly lift the hand, as if to cuff a partner, and the sportive lemurs additionally show a branch-shaking display [103,104]. Cathemeral and diurnal group-living lemurs exhibit 3–22 visual displays, with the largest number of visual signals reported in ring-tailed lemurs [74].

Visual signals are mainly manual or bodily gestures, whereas facial expressions are rare. This fact might be due to morphological constraints because lemurs have less well diversified facial muscles involved in coordination of facial expression than anthropoid primates [105,106]. Facial expressions in lemurs mainly consist of mouth-displays, such as the open-mouth display that is produced during play in ring-tailed lemurs, Coquerel's and Verreaux's sifakas (*Propithecus coquereli*, *P. verreauxi*; figure 1*f*) [107,108]. Yawning is associated with a sleep–wake cycle in ring-tailed lemurs and Verreaux's sifakas, but also with anxiety, for example, after predatory attacks [109] or undecided behaviour during agonistic interactions in ring-tailed lemurs [74]. A grimace or facial grin serves as a submissive signal in ring-tailed and black-and-white ruffed lemurs or Verreaux's sifakas [33,74,110]. Since there is only little information available on the social use of visual signals, we encourage future research to study the use of visual signals in more detail.

By contrast, more stable visual signals such as facial colour complexity in lemurs were associated with ecological factors, with lemurs occurring in less dense, drier habitats exhibiting more complex facial colour patterns [48]. Social factors, such as group size or the number of sympatric species at the family or genus level, did not predict facial colour complexity, even though facial colour patterns can be used for species recognition [111]. However, divergent patterns concerning the importance of social factors in influencing facial colour complexity have been found in New and Old World primates, which nonetheless exhibit convergent patterns regarding the effects of ecological factors [46,47]. In both anthropoid radiations the number of sympatric congeners covaried positively with facial colour complexity, but group size exhibited a negative correlation in New World monkeys and a positive one in Old World monkeys, indicating that the influence of social complexity on the evolution of such stable visual signals appears to be lineage-specific, which should be considered in comprehensive analyses across all primates or mammals [112].

### (c) Environmental factors

Only the number of olfactory sources, but not the number of vocal and visual signals, was influenced by habitat type. The long-lasting components of olfactory signals can be influenced by habitat type, with signals being more easily washed out in habitats with higher rainfall, such as rainforests. Accordingly, lemur occurring in closed and more humid habitats evolved fewer olfactory sources than those occurring in semi-closed and drier habitats. Habitat density, however, may rather impact the transmission of vocal signals and, hence, the acoustic structure of vocalizations [61] as, for example, in frogs [113]. Hence, our classification did not allow us to infer how masking effects of background noise may have impacted the evolution of different vocal signals. Transmission of visual signals is mainly impacted by visibility, which might be constrained in denser habitats or habitats of rapidly moving vegetation, as suggested, for example, in lizards [45,114]. Since the known visual signals in lemurs are mainly used during social interactions, in which the sender and receiver are usually in close proximity, the evolution of these signals was probably not constrained by habitat type.

The number of sympatric species that also produce similar communicative signals during the same activity periods may lead primarily to divergent selection on signal structure to avoid masking interference [59,60]. However, in lemurs, species can be either nocturnal, diurnal or cathemeral [53]. Cathemeral activity is primarily controlled by light availability and has evolved from nocturnal ancestors, presumably presenting a transitory state on the way to the diurnal niche [115,116]. During this evolutionary transition, lemurs occupied not only new ecological [115,116], but also communicative niches, which may have led to signal divergence. However, the number of sympatric species with the same activity pattern did not predict repertoire size in all modalities, suggesting that social factors were primarily responsible for the evolution of larger repertoire sizes. Furthermore, the number of sympatric congeners, with whom hybridization is most likely, did not correlate with repertoire size in any modality either. Since only two genera of lemurs occur in sympatry today, the need for reliable species recognition did apparently not impact signal divergence.

### (d) No trade-offs in signal evolution across modalities

We did not find evidence for a trade-off in signal evolution across modalities. Lemurs that evolved a larger vocal repertoire did so also in the olfactory and visual domains, suggesting that an increase in social complexity evolved in tandem with communicative abilities in general. In the vocal, but also in the olfactory domain, information on individual traits such as sex, identity or condition that are important for mediating social interactions are encoded in both the acoustic structure or chemical composition of these signals [57,76,87,89,117]. Cross-modal individual recognition in vocal and olfactory signals in ring-tailed lemurs supports the notion that both communicative modalities evolved in tandem [118]. However, other individual traits seem to be transmitted only via one modality, such as reproductive state, heterozygosity or relatedness, which are all associated with chemical variation in scrotal gland secretions during the mating season [119,120].

Positive correlations among signal repertoire sizes across species may reflect consistent selection acting similarly on separate modalities, especially if signals convey separate information, resulting in a functional integration among signals [62]. As suggested for the evolution of multiple ornaments in birds, positive correlations among signals would arise when selection favours ‘an integrated whole’ of ornamental traits [62]. In lemurs, enhanced social complexity appeared to be the main driver of selection of signal diversification. Vocal and olfactory signals are associated with similar but also different individual traits, and cross-modal recognition may indicate that selection may have favoured ‘an integrated whole’ across modalities to facilitate communication during social interactions.

### (e) Morphology, brain size and communicative complexity

Repertoire sizes in vocal and visual signals did not correlate with ECV and body mass. Also, the number of olfactory sources did not correlate with body mass, suggesting that biophysical constraints did not impact signal evolution in lemurs. The production of vocalizations in non-human primates is predominantly innate [121,122], whereas the usage and understanding of vocal signals are more flexible [123]. Since vocal repertoire size reflects only the production of vocalizations, which is not associated with behavioural flexibility and cognitive underpinnings therein [123], it is not surprising that we did not find a correlation between vocal repertoire and a proxy of brain size. Hence, complexity indices allowing an operationalization of the flexible use and understanding of non-human primate vocalizations are now required to understand whether vocal communication strategies are related to cognitive abilities.

Also, the number of visual signals was not related to brain size. In Old World primates, which rely more on visual signals such as facial expressions, the size of the primary visual cortex coevolved with facial expression processing [124]. In addition, relative neocortex size, which covaries with group size [125], correlates positively with facial nucleus size as a measure of facial motor control, with a larger nucleus implying more neurons and presumably finer motor control of facial muscles and, hence, more diverse or flexible facial expressions [126,127]. Hence, primary visual cortex or facial nucleus size might have been more appropriate measures than ECV [128,129], but unfortunately these measures are available for only a few lemur species.

In conclusion, we showed that complexity in communication in the vocal, olfactory and visual domains in lemurs coevolved with social complexity, but not with socio-ecological factors such as habitat or the number of sympatric species. Evolutionary changes in social complexity presumably antedated corresponding changes in communicative complexity. These main results support key predictions of the SCHCC. In addition, communicative complexity coevolved across modalities possibly as an ‘an integrated whole’ to facilitate communication during social interactions. The fact that signals and cues—in particular multimodal signals—are perceived by a mix of different sensory modalities [2] supports this notion. Hence, further studies are now indicated to examine whether communicative complexity across modalities also coevolved with sociality and whether sociality influences the evolution of signals in other taxa.

## Data Availability

The datasets supporting this article have been uploaded as part of the electronic supplementary material [130].

## References

[1] Freeberg TM, Dunbar RIM, Ord TJ. 2012 Social complexity as a proximate and ultimate factor in communicative complexity. Phil. Trans. R. Soc. B **367**, 1785-1801. (10.1098/rstb.2011.0213) 22641818 PMC3367695

[2] Peckre L, Kappeler PM, Fichtel C. 2019 Clarifying and expanding the social complexity hypothesis for communicative complexity. Behav. Ecol. Sociobiol. **73**, 11. (10.1007/s00265-018-2605-4)

[3] Freeberg TM, Ord TJ, Dunbar RIM. 2012 The social network and communicative complexity: preface to theme issue. Phil. Trans. R. Soc. B **367**, 1782-1784. (10.1098/rstb.2011.0294) 22641817 PMC3367707

[4] Cheney D, Seyfarth R. 2005 Social complexity and the information acquired during eavesdropping by primates and other animals. In Animal communication networks (ed. PK McGregor), pp. 583-603. Cambridge, UK: Cambridge University Press. (10.1017/CBO9780511610363.030)

[5] Taborsky B, Oliveira RF. 2012 Social competence: an evolutionary approach. Tends Ecol. Evol. **27**, 679-688. (10.1016/j.tree.2012.09.003) 23040461

[6] Sewall KB. 2015 Social complexity as a driver of communication and cognition. Integr. Comp. Biol. **55**, 384-395. (10.1093/icb/icv064) 26078368

[7] Mccomb K, Semple S. 2005 Coevolution of vocal communication and sociality in primates. Biol. Lett. **1**, 381-385. (10.1098/rsbl.2005.0366) 17148212 PMC1626386

[8] Roberts AI, Roberts SGB. 2020 Communicative roots of complex sociality and cognition. Biol. Rev. **95**, 51-73. (10.1111/brv.12553) 31608566

[9] Varela SAM, Teles MC, Oliveira RF. 2020 The correlated evolution of social competence and social cognition. Funct. Ecol. **34**, 332-343. (10.1111/1365-2435.13416)

[10] Dobson SD. 2009 Allometry of facial mobility in anthropoid primates: implications for the evolution of facial expression. Am. J. Phys. Anthropol. **138**, 70-81. (10.1002/ajpa.20902) 18711735

[11] Gustison ML, Roux AL, Bergman TJ. 2012 Derived vocalizations of geladas (*Theropithecus gelada*) and the evolution of vocal complexity in primates. Phil. Trans. R. Soc. B **367**, 1847-1859. (10.1016/0306-4530(89)90065-6) 22641823 PMC3367700

[12] Kavanagh E et al. 2021 Dominance style is a key predictor of vocal use and evolution across nonhuman primates. R. Soc. Open Sci. **8**, 210873. (10.1098/rsos.210873) 34350023 PMC8316807

[13] Rebout N et al. 2020 Tolerant and intolerant macaques show different levels of structural complexity in their vocal communication. Proc. R. Soc. B **287**, 20200439. (10.1098/rspb.2020.0439) PMC7341924 32517610

[14] Roberts SGB, Roberts AI. 2020 Social and ecological complexity is associated with gestural repertoire size of wild chimpanzees. Integr. Zool. **15**, 276-292. (10.1111/1749-4877.12423) 31773892 PMC7383666

[15] DelBarco-Trillo J, Sacha CR, Dubay GR, Drea CM. 2012 Eulemur, me lemur: the evolution of scent-signal complexity in a primate clade. Phil. Trans. R. Soc. B **367**, 1909-1922. (10.1098/rstb.2011.0225) 22641829 PMC3367706

[16] Wilkinson GS et al. 2019 Kinship, association, and social complexity in bats. Behav. Ecol. Sociobiol. **73**, 7. (10.1007/s00265-018-2608-1)

[17] Blumstein DT, Armitage KB. 1997 Does sociality drive the evolution of communicative complexity? A comparative test with ground-dwelling sciurid alarm calls. Am. Nat. **150**, 179-200. (10.1086/286062) 18811281

[18] Pollard KA, Blumstein DT. 2011 Social group size predicts the evolution of individuality. Curr. Biol. **21**, 413-417. (10.1016/j.cub.2011.01.051) 21333537

[19] Leighton GM. 2017 Cooperative breeding influences the number and type of vocalizations in avian lineages. Proc. R. Soc. B **284**, 20171508. (10.1098/rspb.2017.1508) PMC5740270 29187625

[20] Leighton GM, Birmingham T. 2021 Multiple factors affect the evolution of repertoire size across birds. Behav. Ecol. **32**, 380-385. (10.1093/beheco/araa139)

[21] Freeberg TM. 2016 Social complexity can drive vocal complexity: group size influences vocal information in Carolina chickadees. Psychol. Sci. **17**, 557-561. (10.1111/j.1467-9280.2006.01743.x) 16866738

[22] Soma M, Brumm H. 2020 Group living facilitates the evolution of duets in barbets. Biol. Lett. **16**, 20200399. (10.1098/rsbl.2020.0399) 32842898 PMC7480159

[23] Baeckens S, Whiting MJ. 2021 Investment in chemical signaling glands facilitates the evolution of sociality in lizards. Proc. R. Soc. B **288**, 20202438. (10.1098/rspb.2020.2438) PMC7935108 33593182

[24] Ord TJ, Blumstein DT, Evans CS. 2002 Ecology and signal evolution in lizards. Biol. Linn. Soc. Lond. **77**, 127-148. (10.1046/j.1095-8312.2002.00100.x)

[25] Wittwer B, Hefetz A, Simon T, Murphy LEK, Elgar MA, Pierce NE, Kocher SD. 2017 Solitary bees reduce investment in communication compared with their social relatives. Proc. Natl Acad. Sci. USA **114**, 6569-6574. (10.1073/pnas.1620780114) 28533385 PMC5488929

[26] Patricelli GL, Hebets EA. 2016 New dimensions in animal communication: the case for complexity. Curr. Opin. Behav. Sci. **12**, 80-89. (10.1016/j.cobeha.2016.09.011)

[27] Fischer J, Wadewitz P, Hammerschmidt K. 2016 Structural variability and communicative complexity in acoustic communication. Anim. Behav. **134**, 229-237. (10.1016/j.anbehav.2016.06.012)

[28] Kappeler PM. 2019 A framework for studying social complexity. Behav. Ecol. Sociobiol. **73**, 13. (10.1007/s00265-018-2601-8)

[29] Hobson EA, Ferdinand V, Kolchinsky A, Garland J. 2019 Rethinking animal social complexity measures with the help of complex systems concepts. Anim. Behav. **155**, 287-296. (10.1016/j.anbehav.2019.05.016)

[30] Morrison RE, Eckardt W, Stoinski TS, Brent LJN. 2020 Comparing measures of social complexity: larger mountain gorilla groups do not have a greater diversity of relationships. Proc. R. Soc. B **287**, 20201026. (10.1098/rspb.2020.1026) PMC7423670 33043865

[31] Dunbar RIM, Shultz S. 2021 Social complexity and the fractal structure of group size in primate social evolution. Biol. Rev. **96**, 1889-1906. (10.1111/brv.12730) 33945202

[32] Ord TJ, Garcia-Porta J. 2012 Is sociality required for the evolution of communicative complexity? Evidence weighed against alternative hypotheses in diverse taxonomic groups. Phil. Trans. R. Soc. B **367**, 1811-1828. (10.1098/rstb.2011.0215) 22641820 PMC3367697

[33] Jolly A. 1966 Lemur social behavior and primate intelligence. Science **153**, 501-506. (10.1126/science.153.3735.501) 5938775

[34] Waal Fd, Luttrell LM. 1985 The formal hierarchy of rhesus macaques: an investigation of the bared-teeth display. Am. Primatol. **9**, 73-85. (10.1002/ajp.1350090202) 32102494

[35] Gillooly JF, Ophir AG. 2010 The energetic basis of acoustic communication. Proc. R. Soc. B **277**, 1325-1331. (10.1098/rspb.2009.2134) PMC2871947 20053641

[36] Charlton BD, Reby D. 2016 The evolution of acoustic size exaggeration in terrestrial mammals. Nat. Commun. **7**, 12739. (10.1038/ncomms12739) 27598835 PMC5025854

[37] Kang C, Zahiri R, Sherratt TN. 2017 Body size affects the evolution of hidden colour signals in moths. Proc. R. Soc. B **284**, 20171287. (10.1098/rspb.2017.1287) PMC5577493 28855366

[38] Loeffler-Henry K, Kang C, Sherratt TN. 2019 Consistent associations between body size and hidden contrasting color signals across a range of insect taxa. Am. Nat. **194**, 28-37. (10.1086/703535) 31251647

[39] Ord TJ, Blumstein DT. 2002 Size constraints and the evolution of display complexity: why do large lizards have simple displays? Biol. J. Linn. Soc. Lond. **76**, 145-161. (10.1111/j.1095-8312.2002.tb01721.x)

[40] Campbell P, Pasch B, Pino JL, Crino OL, Phillips M, Phelps SM. 2010 Geographic variation in the songs of neotropical singing mice: testing the relative importance of drift and local adaptation. Evolution **64**, 1955-1977. (10.1111/j.1558-5646.2010.00962.x) 20148958

[41] Cantor M, Shoemaker LG, Cabral RB, Flores CO, Varga M, Whitehead H. 2015 Multilevel animal societies can emerge from cultural transmission. Nat. Commun. **6**, 8091. (10.1038/ncomms9091) 26348688 PMC4569709

[42] Sun C, Jiang T, Gu H, Guo X, Zhang C, Gong L, Shi B, Feng J. 2020 Geographical variation of social calls and vocal discrimination in male Himalayan leaf-nosed bats. Anim. Behav. **170**, 15-26. (10.1016/j.anbehav.2020.10.003)

[43] Halfwerk W, Bot S, Buikx J, Velde Mvd, Komdeur J, Cate Ct, Slabbekoorn H. 2011 Low-frequency songs lose their potency in noisy urban conditions. Proc. Natl Acad. Sci. USA **108**, 14 549-14 554. (10.1073/pnas.1109091108) PMC3167545 21876157

[44] Peters RA. 2008 Environmental motion delays the detection of movement-based signals. Biol. Lett. **4**, 2-5. (10.1098/rsbl.2007.0422) 17971317 PMC2412918

[45] Ord TJ, Stamps JA. 2008 Alert signals enhance animal communication in ‘noisy’ environments. Proc. Natl Acad. Sci. USA **105**, 18 830-18 835. (10.1073/pnas.0807657105) PMC2596256 19033197

[46] Santana SE, Alfaro JL, Alfaro ME. 2012 Adaptive evolution of facial colour patterns in neotropical primates. Proc. R. Soc. B **279**, 2204-2211. (10.1098/rspb.2011.2326) PMC3321701 22237906

[47] Alfaro JL, Noonan A, Alfaro ME, Santana SE. 2013 Adaptive response to sociality and ecology drives the diversification of facial colour patterns in catarrhines. Nat. Commun. **4**, 1-7. (10.1038/ncomms3765) 24212362

[48] Rakotonirina H, Kappeler PM, Fichtel C. 2017 Evolution of facial color pattern complexity in lemurs. Sci. Rep. **7**, 15181. (10.1038/s41598-017-15393-7) 29123214 PMC5680244

[49] Yoder AD, Cartmill M, Ruvolo M, Smith K, Vilga R. 1996 Ancient single origin for Malagasy primates. Proc. Natl Acad. Sci. USA **93**, 5122-5126. (10.1073/pnas.93.10.5122) 8643538 PMC39417

[50] Kappeler PM, Pozzi L. 2019 Evolutionary transitions toward pair living in nonhuman primates as stepping stones toward more complex societies. Sci. Adv. **5**, eaay1276. (10.1126/sciadv.aay1276) 32064318 PMC6989303

[51] Kappeler PM. 1997 Determinants of primate social organization: comparative evidence and new insights from Malagasy lemurs. Biol. Rev. Camb. Philos. Soc. **72**, 111-151. (10.1017/s0006323196004999) 9116164

[52] Kappeler PM, Fichtel C. 2015 Eco-evo-devo of the lemur syndrome: did adaptive behavioral plasticity get canalized in a large primate radiation? Front. Zool. **12**, S15. (10.1186/1742-9994-12-s1-s15) 26816515 PMC4722368

[53] Santini L, Rojas D, Donati G. 2015 Evolving through day and night: origin and diversification of activity pattern in modern primates. Behav. Ecol. **26**, 789-796. (10.1093/beheco/arv012)

[54] Fichtel C. 2012 Predation. In The evolution of primate societies (eds J Mitani, J Call, PM Kappeler, R Palombit, J Silk), pp. 169-194. Chicago, IL: Chicago University Press.

[55] Fichtel C. 2016 Predation in the dark: antipredator strategies of Cheirogaleidae and other nocturnal primates. In The dwarf and mouse lemurs of Madagascar. Biology, behavior and conservation biogeography of the cheirogaleidae (eds S Lehman, U Radespiel, E Zimmermann), pp. 366-380. Cambridge, UK: Cambridge University Press.

[56] Muldoon KM, Goodman SM. 2010 Ecological biogeography of Malagasy non-volant mammals: community structure is correlated with habitat. J. Biogeogr. **37**, 1144-1159. (10.1111/j.1365-2699.2010.02276.x)

[57] Drea CM. 2020 Design, delivery and perception of condition-dependent chemical signals in strepsirrhine primates: implications for human olfactory communication. Phil. Trans. R. Soc. B **375**, 20190264. (10.1098/rstb.2019.0264) 32306880 PMC7209935

[58] Colquhoun IC. 2011 A review and interspecific comparison of nocturnal and cathemeral strepsirhine primate olfactory behavioural ecology. Int. J. Zool. **2011**, 1-11. (10.1038/nature05402)

[59] Endler J. 1993 Some general comments on the evolution and design of animal communication systems. Phil. Trans. R. Soc. B **340**, 215-225. (10.1098/rstb.1993.0060) 8101656

[60] Wilkins MR, Seddon N, Safran RJ. 2013 Evolutionary divergence in acoustic signals: causes and consequences. Trends Ecol. Evol. **28**, 156-166. (10.1016/j.tree.2012.10.002) 23141110

[61] Waser PM, Brown CH. 1986 Habitat acoustics and primate communication. Am. J. Primatol. **10**, 135-154. (10.1002/ajp.1350100205) 31979490

[62] Ligon RA, Diaz CD, Morano JL, Troscianko J, Stevens M, Moskeland A, Laman TG, Scholes E. 2018 Evolution of correlated complexity in the radically different courtship signals of birds-of-paradise. PLoS Biol. **16**, e2006962. (10.1371/journal.pbio.2006962) 30457985 PMC6245505

[63] Roberts AI, Chakrabarti A, Roberts SGB. 2019 Gestural repertoire size is associated with social proximity measures in wild chimpanzees. Am. J. Primatol. **81**, e22954-15. (10.1002/ajp.22954) 30706956

[64] Isler K, Kirk EC, Miller JMA, Albrecht GA, Gelvin BR, Martin RD. 2008 Endocranial volumes of primate species: scaling analyses using a comprehensive and reliable data set. J. Hum. Evol. **55**, 967-978. (10.1016/j.jhevol.2008.08.004) 18817943

[65] Paradis E, Schliep K. 2019 ape 5.0: an environment for modern phylogenetics and evolutionary analyses in R. Bioinformatics **35**, 526-528. (10.1093/bioinformatics/bty633) 30016406

[66] Orme D, Freckleton R, Thomas G, Petzoldt T, Fritz S, Isaac N, Pearse W. 2018 caper: Comparative analyses of phylogenetics and evolution in R. R package version 1.0.1. See https://CRAN.R-project.org/package=caper.

[67] Alfaro ME, Santini F, Brock C, Alamillo H, Dornburg A, Rabosky D, Carneval G, Harmon L. 2009 Nine exceptional radiations plus high turnover explain species diversity in jawed vertebrates. J. Biol. Linn. Soc. Lond. **106**, 13 410-13 414. (10.1073/pnas.0811087106) PMC2715324 19633192

[68] R Core Team. 2020 R: a language and environment for statistical computing. Vienna, Austria: R Foundation for Statistical Computing.

[69] Upham NS, Esselstyn JA, Jetz W. 2019 Inferring the mammal tree: species-level sets of phylogenies for questions in ecology, evolution, and conservation. PLoS Biol. **17**, e3000494. (10.1371/journal.pbio.3000494) 31800571 PMC6892540

[70] Mundry R. 2014 Statistical issues and assumptions of phylogenetic generalized least squares. In Modern phylogenetic comparative methods and their application in evolutionary biology (ed. LZ Garamszegi), pp. 131-153. Berlin, Germany: Springer.

[71] Beaulieu JM, Jhwueng D, Boettiger C, O'Meara BC. 2012 Modeling stabilizing selection: expanding the Ornstein–Uhlenbeck model of adaptive evolution. Evolution **66**, 2369-2383. (10.1111/j.1558-5646.2012.01619.x) 22834738

[72] Dunbar RI, Shultz S. 2010 Bondedness and sociality. Behaviour **147**, 775-803. (10.1163/000579510×501151)

[73] Kappeler P, Heymann E. 1996 Nonconvergence in the evolution of primate life history and socio-ecology. Biol. J. Linn. Soc. Lond. **59**, 297-326. (10.1111/j.1095-8312.1996.tb01468.x)

[74] Pereira M, Kappeler PM. 1997 Divergent systems of agonstic behaviour in lemurid primates. Behaviour **134**, 225-274. (10.1163/156853997X00467)

[75] Fichtel C, Hilgartner R. 2013 Noises in the dark: vocal communication in nocturnal pair-living primates. In Leaping ahead: advances in prosimian biology (eds J Master, M Gamba, F Génin), pp. 297-304. New York, NY: Springer.

[76] Pflüger FJ, Fichtel C. 2012 On the function of redfronted lemur's close calls. Anim. Cogn. **15**, 823-831. (10.1007/s10071-012-0507-9) 22573307 PMC3424289

[77] Macedonia J. 1990 What is communicated in the antipredator calls of lemurs: evidence from playback experiments with ringtailed and ruffed lemurs. Etholgy **86**, 177-190. (10.1111/j.1439-0310.1990.tb00428.x)

[78] Pereira ME, Macedonia JM. 1991 Ringtailed lemur anti-predator calls denote predator class, not response urgency. Anim. Behav. **41**, 543-544. (10.1016/s0003-3472(05)80861-9)

[79] Fichtel C, Kappeler P. 2002 Anti-predator behavior of group-living Malagasy primates: mixed evidence for a referential alarm call system. Behav. Ecol. Sociobiol. **51**, 262-275. (10.1007/s00265-001-0436-0)

[80] Rahlfs M, Fichtel C. 2010 Anti-predator behaviour in a nocturnal primate, the Grey Mouse Lemur (*Microcebus murinus*). Ethology **116**, 429-439. (10.1111/j.1439-0310.2010.01756.x)

[81] Fichtel C. 2020 Monkey alarm calling: it ain't all referential, or is it? Anim. Behav. Cogn. **7**, 101-107. (10.26451/abc.07.02.04.2020)

[82] Trillmich J, Fichtel C, Kappeler P. 2004 Coordination of group movements in wild Verreaux's sifakas (*Propithecus verreauxi*). Behaviour **141**, 1103-1120. (10.1163/1568539042664579)

[83] Braune P, Schmidt S, Zimmermann E. 2005 Spacing and group coordination in a nocturnal primate, the golden brown mouse lemur (*Microcebus ravelobensis*): the role of olfactory and acoustic signals. Behav. Ecol. Sociobiol. **58**, 587-596. (10.1007/s00265-005-0944-4)

[84] Sperber AL, Werner LM, Kappeler PM, Fichtel C. 2017 Grunt to go—vocal coordination of group movements in redfronted lemurs. Ethology **123**, 894-905. (10.1111/eth.12663)

[85] Bolt LM. 2020 Affiliative contact calls during group travel: chirp and wail vocalization use in the male ring-tailed lemur (*Lemur catta*). Folia Primatol. **91**, 575-594. (10.1159/000508808) 32756063

[86] Zimmermann E. 2003 Castration affects the emission of an ultrasonic vocalization in a nocturnal primate, the grey mouse lemur (*Microcebus murinus*). Physiol. Behav. **60**, 693-697. (10.1016/0031-9384(96)81674-x) 8873238

[87] Buesching CD, Heistermann M, Folia JH. 1998 Multimodal oestrus advertisement in a small nocturnal prosimian, *Microcebus murinus*. Folia Primatol. **69**, 295-308. (10.1159/000052718)

[88] Walker-Bolton AD, Parga JA. 2017 ‘Stink flirting’ in ring-tailed lemurs (*Lemur catta*): male olfactory displays to females as honest, costly signals. Am. J. Primatol. **79**, e22724. (10.1002/ajp.22724) 29140563

[89] Fichtel C, Kappeler PM, Perret M, Huchard E, Henry P-Y. 2021 Honest signaling in mouse lemur vocalizations? Int. J. Primatol. 1-22. (10.1007/s10764-021-00265-9)

[90] Bolt LM. 2013 The function of howling in the ring-tailed lemur (*Lemur catta*). Int. J. Primatol. **34**, 157-169. (10.1007/s10764-012-9654-8)

[91] Rakotonirina H, Kappeler PM, Fichtel C. 2016 The role of acoustic signals for species recognition in redfronted lemurs (*Eulemur rufifrons*). BMC Evol. Biol. **16**, 100. (10.1186/s12862-016-0677-1) 27175922 PMC4866039

[92] Kulahci IG, Rubenstein DI, Ghazanfar AA. 2015 Lemurs groom-at-a-distance through vocal networks. Anim. Behav. **110**, 179-186. (10.1016/j.anbehav.2015.09.016)

[93] Freeberg TM, Gentry KE, Sieving KE, Lucas JR. 2019 On understanding the nature and evolution of social cognition: a need for the study of communication. Anim. Behav. **155**, 279-286. (10.1016/j.anbehav.2019.04.014)

[94] Ramsier MA, Cunningham AJ, Finneran JJ, Dominy NJ. 2012 Social drive and the evolution of primate hearing. Phil. Trans. R. Soc. B **367**, 1860-1868. (10.1098/rspb.1995.0029) 22641824 PMC3367701

[95] Silk RMSDLCJB, Seyfarth R, Cheney D. 2016 Strategic use of affiliative vocalizations by wild female baboons. PLoS ONE **11**, 1-10. (10.1371/journal.pone.0163978) PMC5081171 27783705

[96] Gosset D, Fornasieri I, Roeder J-J. 2001 Acoustic structure and contexts of emission of vocal signals by black lemurs. Evol. Commun. **4**, 225-251. (10.1075/eoc.4.2.06gos)

[97] Pozzi L, Gamba M, Giacoma C. 2010 The use of artificial neural networks to classify primate vocalizations: a pilot study on black lemurs. Am. J. Primatol. **72**, 337-348. (10.1002/ajp.20786) 20034021

[98] Gamba M, Friard O, Riondato I, Righini R, Colombo C, Miaretsoa L, Torti V, Nadhurou B, Giacoma C. 2015 Comparative analysis of the vocal repertoire of *Eulemur*: a dynamic time warping approach. Int. J. Primatol. **36**, 894-910. (10.1007/s10764-015-9861-1)

[99] Eschmann C. 2019 A comparison of *Eulemur* social systems and vocal communication during the mating season: implications for the speciation and conservation of blue-eyed black lemurs and black lemurs. PhD thesis, University of Bristol, UK.

[100] DelBarco-Trillo J, Burkert BA, Goodwin TE, Drea CM. 2011 Night and day: the comparative study of strepsirrhine primates reveals socioecological and phylogenetic patterns in olfactory signals. J. Evol. Biol. **24**, 82-98. (10.1111/j.1420-9101.2010.02145.x) 21091564

[101] Scordato E, Dubay G, Drea C. 2007 Chemical composition of scent marks in the ringtailed lemur (*Lemur catta*): glandular differences, seasonal variation, and individual signatures. Chem. Senses **32**, 493-504. (10.1093/chemse/bjm018) 17488747

[102] Peckre LR, Michiels A, Socias-Martínez L, Kappeler PM, Fichtel C. 2022 Sex differences in audience effects on anogenital scent marking in the red-fronted lemur. Scientific Reports **12**, 1-16. (10.1038/s41598-022-08861-2) 35347156 PMC8960772

[103] Rasoloharijaona S, Randrianambinina B, Braune P, Zimmermann E. 2006 Loud calling, spacing, and cohesiveness in a nocturnal primate, the Milne Edwards' sportive lemur (*Lepilemur edwardsi*). Am. J. Phys. Anthropol. **129**, 591-600. (10.1002/ajpa.20342) 16331660

[104] Hilgartner R. 2007 Living apart together: pair-living in red-tailed sportive lemurs (*Lepilemur ruficaudatus*). PhD thesis, University of Göttingen, Germany.

[105] Andrew RJ. 1962 The origin and evolution of the calls and facial expressions of the primates. Behaviour **20**, 1-107. (10.1163/156853963X00220)

[106] Burrows AM, Smith TD. 2003 Muscles of facial expression in *Otolemur*, with a comparison to Lemuroidea. Anat. Rec. A Discov. Mol. Cell Evol. Biol. **274A**, 827-836. (10.1002/ar.a.10093) 12923893

[107] Palagi E, Norscia I, Spada G. 2014 Relaxed open mouth as a playful signal in wild ring-tailed lemurs. Am. J. Primatol. **76**, 1074-1083. (10.1002/ajp.22294) 24810169

[108] Malalaharivony H, Kappeler P, Fichtel C. 2021 Infant development and maternal care in wild Verreaux's sifaka (*Propithecus verreauxi*). Int. J. Primatol. **42**, 933-960. (10.1007/s10764-021-00255-x)

[109] Zannella A, Norscia I, Stanyon R, Palagi E. 2015 Testing yawning hypotheses in wild populations of two strepsirrhine species: *Propithecus verreauxi* and *Lemur catta*. Am. J. Primatol. **77**, 1207-1215. (10.1002/ajp.22459) 26317594

[110] Pereira ME, Seeligson ML, Macedonia JM. 1988 The behavioral repertoire of the black-and-white ruffed lemur, *Varecia variegata variegata* (Primates: Lemuridae). Folia Primatol. **51**, 1-32. (10.1159/000156353) 3251818

[111] Rakotonirina H, Kappeler PM, Fichtel C. 2018 The role of facial pattern variation for species recognition in red-fronted lemurs (*Eulemur rufifrons*). BMC Evol. Biol. **18**, 19. (10.1186/s12862-018-1126-0) 29433448 PMC5809826

[112] Kappeler P. 2021 Complex adaptations in a multivariate trait: a comment on Caro *et al*. Behav. Ecol. **32**, 569-570. (10.1093/beheco/arab037)

[113] Feng AS, Narins PM, Xu C-H, Lin W-Y, Yu Z-L, Qiu Q, Xu Z-M, Shen J-X. 2006 Ultrasonic communication in frogs. Nature **440**, 333-336. (10.1038/nature04416) 16541072

[114] Ord TJ, Martins EP. 2006 Tracing the origins of signal diversity in anole lizards: phylogenetic approaches to inferring the evolution of complex behaviour. Anim. Behav. **71**, 1411-1429. (10.1016/j.anbehav.2005.12.003)

[115] Kappeler PM, Erkert HG. 2003 On the move around the clock: correlates and determinants of cathemeral activity in wild redfronted lemurs (*Eulemur fulvus rufus*). Behav. Ecol. Sociobiol. **54**, 359-369. (10.1007/s00265-003-0652-x)

[116] Schaik CP, Kappeler PM. 1996 The social systems of gregarious lemurs: lack of convergence with anthropoids due to evolutionary disequilibrium? Ethology **102**, 915-941. (10.1111/j.1439-0310.1996.tb01171.x)

[117] Leliveld LMC, Scheumann M, Zimmermann E. 2011 Acoustic correlates of individuality in the vocal repertoire of a nocturnal primate (*Microcebus murinus*). J. Acoust. Soc. Am. **129**, 2278-2288. (10.1121/1.3559680) 21476683

[118] Kulahci IG, Drea CM, Rubenstein DI, Ghazanfar AA. 2014 Individual recognition through olfactory–auditory matching in lemurs. Proc. R. Soc. B **281**, 20140071. (10.1098/rspb.2014.0071) PMC4043086 24741013

[119] Charpentier MJE, Prugnolle F, Gimenez O, Widdig A. 2008 Genetic heterozygosity and sociality in a primate species. Behav. Genet. **38**, 151-158. (10.1007/s10519-008-9191-6) 18293079

[120] Charpentier MJE, Crawford JC, Boulet M, Drea CM. 2010 Message ‘scent’: lemurs detect the genetic relatedness and quality of conspecifics via olfactory cues. Anim. Behav. **80**, 101-108. (10.1016/j.anbehav.2010.04.005)

[121] Hammerschmidt K, Freudenstein T, Jürgens U. 2001 Vocal development in squirrel monkeys. Behaviour **138**, 1179-1204. (10.1163/156853901753287190)

[122] Seyfarth RM, Cheney DL. 2003 Signalers and receivers in animal communication. Annu. Rev. Psychol. **54**, 145-173. (10.1146/annurev.psych.54.101601.145121) 12359915

[123] Cheney DL, Seyfarth RM. 2018 Flexible usage and social function in primate vocalizations. Proc. Natl Acad. Sci. USA **115**, 201717572. (10.1073/pnas.1717572115) PMC5834704 29432157

[124] Dobson SD, Sherwood CC. 2011 Correlated evolution of brain regions involved in producing and processing facial expressions in anthropoid primates. Biol. Lett. **7**, 86-88. (10.1098/rsbl.2010.0427) 20591852 PMC3030864

[125] Dunbar R. 1995 Neocortex size and group size in primates: a test of the hypothesis. J. Hum. Evol. **28**, 287-296. (10.1006/jhev.1995.1021)

[126] Sherwood CC, Hof PR, Holloway RL, Semendeferi K, Gannon PJ, Frahm HD, Zilles K. 2005 Evolution of the brainstem orofacial motor system in primates: a comparative study of trigeminal, facial, and hypoglossal nuclei. J. Hum. Evol. **48**, 45-84. (10.1016/j.jhevol.2004.10.003) 15656936

[127] Dobson SD. 2012 Coevolution of facial expression and social tolerance in macaques. Am. J. Primatol. **74**, 229-235. (10.1002/ajp.21991) 24006541

[128] Healy SD, Rowe C. 2013 Costs and benefits of evolving a larger brain: doubts over the evidence that large brains lead to better cognition. Anim. Behav. **86**, e1-e3. (10.1016/j.anbehav.2013.05.017)

[129] Barrett L, Henzi SP, Barton RA. 2022 Experts in action: why we need an embodied social brain hypothesis. Phil. Trans. R. Soc. B **377**, 20200533. (10.1098/rstb.2020.0533) 34957849 PMC8710874

[130] Fichtel C, Kappeler PM. 2022 Data from: Coevolution of social and communicative complexity in lemurs. Figshare. (10.6084/m9.figshare.c.6060616) PMC9358322 35934963

